# Supplementary figures and images for: Validated Methods for Inactivation of Tick-Borne Encephalitis Virus Compatible with Immune-Based and Enzymatic Downstream Analyses
Source: Viruses. 2025 Jun 3;17(6):810. doi: 10.3390/v17060810 (PMC12197580; doi:10.3390/v17060810)

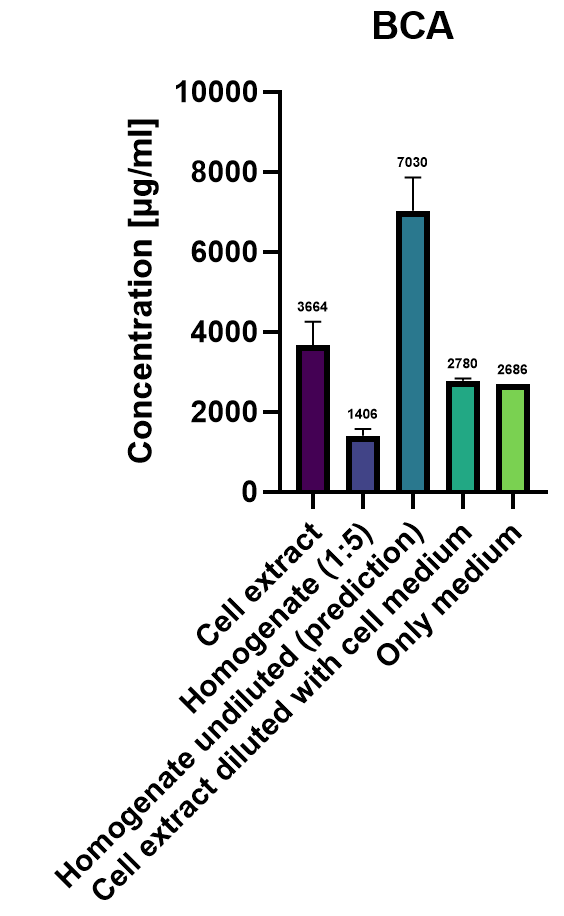

Supplement: Supplementary file 1 [file viruses-17-00810-s001.zip › Supplementary Figure 1 - Bicinchoninic acid (BCA) assay.PNG]

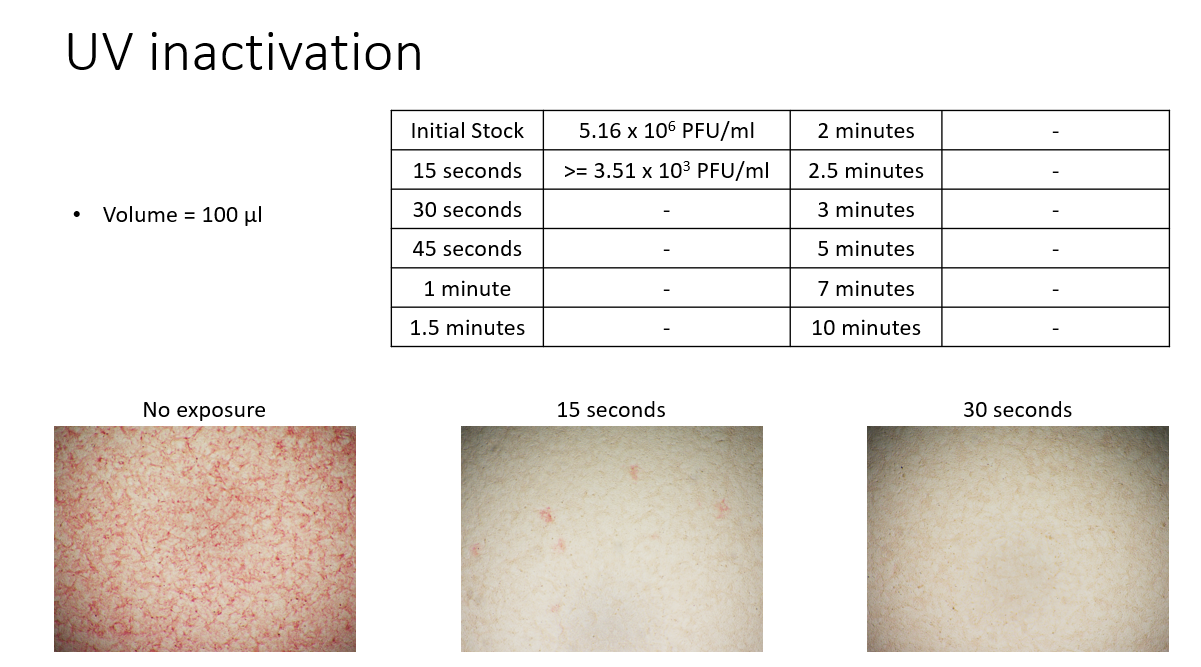

Supplement: Supplementary file 1 [file viruses-17-00810-s001.zip › Supplementary Figure 2 - UV inactivation (TCID50).PNG]

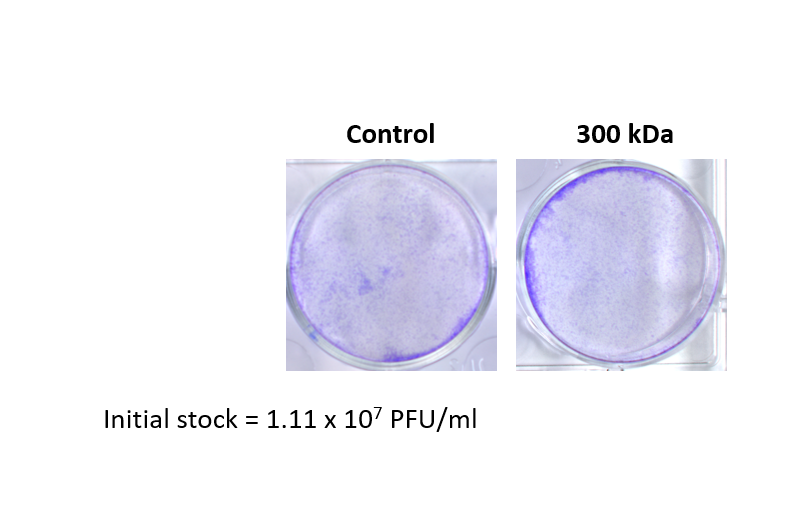

Supplement: Supplementary file 1 [file viruses-17-00810-s001.zip › Supplementary Figure 3 - 300 kDa filtration.png]
